# 3,5-Dibromo-2-hydroxy­benzaldehyde

**DOI:** 10.1107/S1600536808008726

**Published:** 2008-04-04

**Authors:** Ying Fan, Wei You, Hui-Fen Qian, Jian-Lan Liu, Wei Huang

**Affiliations:** a College of Sciences, Nanjing University of Technology, Nanjing 210009, People’s Republic of China; b State Key Laboratory of Coordination Chemistry, Coordination Chemistry Institute, Nanjing University-Jinchuan Group Ltd Joint Laboratory of Metal Chemistry, School of Chemistry and Chemical Engineering, Nanjing University, Nanjing 210093, People’s Republic of China

## Abstract

The title compound, C_7_H_4_Br_2_O_2_, exhibits a layer packing structure *via* weak π–π stacking inter­actions [centroid–centroid distances between adjacent aromatic rings are 4.040 (8) and 3.776 (7) Å]. Mol­ecules in each layer are linked by inter­molecular O—H⋯O hydrogen bonding and Br⋯Br inter­actions [3.772 (4) Å]. There are two mol­ecules in the asymmetric unit.

## Related literature

For related compounds, see Harkat *et al.* (2008 ▶); Lu *et al.* (2006 ▶); Duan *et al.* (2007 ▶); Zhang *et al.* (2007 ▶).
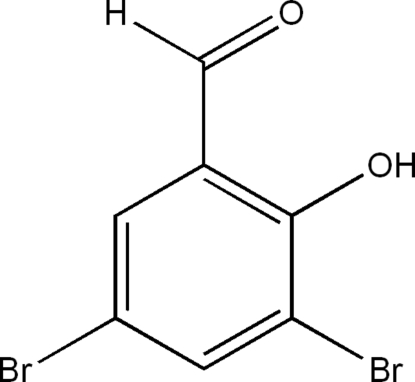


## Experimental

### 

#### Crystal data

C_7_H_4_Br_2_O_2_
*M* *_r_* = 279.92
Monoclinic, 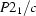

*a* = 16.474 (8) Å
*b* = 14.025 (10) Å
*c* = 7.531 (7) Å
β = 103.212 (2)°
*V* = 1694 (2) Å^3^
*Z* = 8
Mo *K*α radiation
μ = 9.52 mm^−1^
*T* = 291 (2) K
0.10 × 0.10 × 0.10 mm

#### Data collection

Bruker SMART CCD area-detector diffractometer
Absorption correction: multi-scan (*SADABS*; Bruker, 2000 ▶) *T*_min_ = 0.450, *T*_max_ = 0.450 (expected range = 0.386–0.386)
8777 measured reflections
3328 independent reflections
1670 reflections with *I* > 2σ(*I*)
*R*_int_ = 0.109

#### Refinement

*R*[*F* ^2^ > 2σ(*F* ^2^)] = 0.045
*wR*(*F* ^2^) = 0.105
*S* = 0.79
3328 reflections
202 parameters
H-atom parameters constrained
Δρ_max_ = 0.67 e Å^−3^
Δρ_min_ = −0.55 e Å^−3^

### 

Data collection: *SMART* (Bruker, 2000 ▶); cell refinement: *SAINT* (Bruker, 2000 ▶); data reduction: *SAINT*; program(s) used to solve structure: *SHELXTL* (Sheldrick, 2008 ▶); program(s) used to refine structure: *SHELXTL*; molecular graphics: *SHELXTL*; software used to prepare material for publication: *SHELXTL*.

## Supplementary Material

Crystal structure: contains datablocks global, I. DOI: 10.1107/S1600536808008726/at2547sup1.cif

Structure factors: contains datablocks I. DOI: 10.1107/S1600536808008726/at2547Isup2.hkl

Additional supplementary materials:  crystallographic information; 3D view; checkCIF report

## Figures and Tables

**Table 1:** Hydrogen-bond geometry (Å, °)

*D*—H⋯*A*	*D*—H	H⋯*A*	*D*⋯*A*	*D*—H⋯*A*
O2—H2⋯O1	0.82	1.94	2.660 (6)	146
O4—H4*A*⋯O3	0.82	2.01	2.713 (6)	143
O4—H4*A*⋯O1^i^	0.82	2.29	2.863 (6)	128
C7—H7⋯O3^ii^	0.93	2.55	3.122 (8)	120

Symmetry codes: (i) 
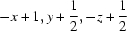
; (ii) 
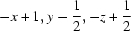
.

**Fig. 1.**
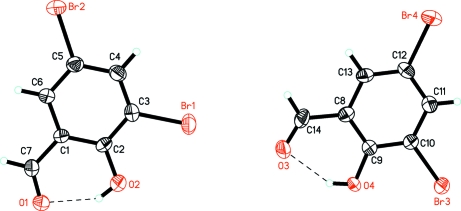
An ORTEP drawing of the title compound with the atom-numbering scheme. Displacement ellipsoids are drawn at the 30% probability level.

**Fig. 2.**
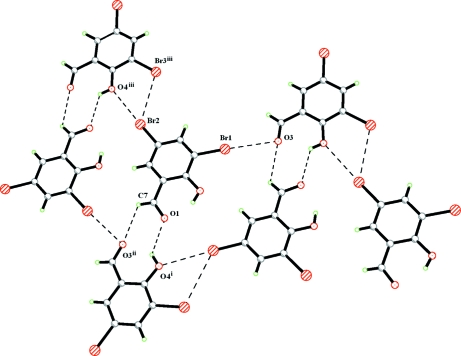
A perspective view of the intralayer intermolecular hydrogen-bond contacts among molecules in the title compound. Hydrogen bonds and Br–Br interactions are shown as dashed lines. [Symmetry codes: (i) -x + 1, y + 1/2, -z + 1/2; (ii) - x + 1,-1/2 + y, 1/2 - z; (iii) x, -1 + y, z.]

**Fig. 3.**
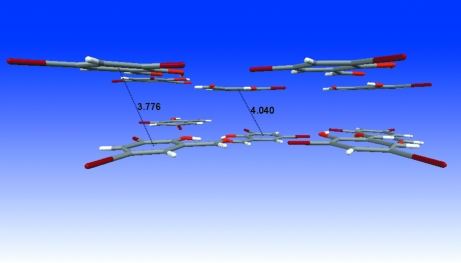
A perspective view of the interlayer π–π stacking interactions together with the centroid–centroid contacts.

## supplementary crystallographic information

### Comment

Slicylaldehyde and its derivatives are an important class of compounds which can be used in a variety of studies such as organic synthesis, catalyst, drug design, spicery industry and life science and so on (Harkat *et al.*, 2008). In the past few decades, a continuing attention has been drawn to the derivatives of the salicylaldehyde and their metal complexes for the investigation of luminescent properties which could be finely tuned by different substituent groups bonded to the phenolic ring (Lu *et al.*, 2006; Duan *et al.*, 2007; Zhang *et al.*, 2007). In this paper, we report the X-ray structure of 3,5-dibromo-2-hydroxybenzaldehyde, (I).

The molecular structure of (I) is illustrated in Fig. 1. There are two crystallographically independent molecules in the asymmetric unit, and both of them are essentially planar with the dihedral angle of 1.82 (6)°.

The C—H···O and O—H···O hydrogen bonding interactions contribute to the stabilizations of the molecular and crystal structures (Fig. 2 and Table 1). A layer packing structure is formed with the mean interlayer separation of 4.040 (8) and 3.776 (7) Å for two sets of molecules. The centeroid-to-centeriod separations between the adjacent aromatic rings are 4.040 (8) and 3.776 (7) Å, respectively (Fig. 3), indicative of weak π–π stacking interactions.

### Experimental

The title compound was obtained as received. Single crystals suitable for X-ray diffraction measurement were formed after 5 days in ethyl acetate by slow evaporation at room temperature in air. Analysis calculated for C_7_H_4_O_4_Br_2_: C 30.04, H 1.44%. Found: C 30.08, H 1.39%. FT—IR (KBr pellets, cm^-1^): 3180(*m*), 3069(*m*), 1681(*versus*), 1662(*versus*), 1597(*m*), 1448(*s*), 1408(*s*), 1281(*versus*), 1198(*s*), 1151(*m*), 1134(*m*), 1098(*m*), 919(*s*), 877(*s*), 712(*m*) and 677(*s*).

### Refinement

The H atoms bonded with carbon atoms were placed in geometrically idealized positions (C—H = 0.93 Å and O—H = 0.82 Å) and refined as riding atoms, with *U*_iso_(H) = 1.2*U*_eq_(C) and *U*_iso_(H) = 1.5*U*_eq_(O).

### Crystal data

**Table:**

C_7_H_4_Br_2_O_2_	*F*_000_ = 1056
*M**_r_* = 279.92	*D*_x_ = 2.195 Mg m^−^^3^
Monoclinic, *P*2_1_/*c*	Mo *K*α radiation λ = 0.71073 Å
Hall symbol: -P 2ybc	Cell parameters from 1688 reflections
*a* = 16.474 (8) Å	θ = 2.9–22.8º
*b* = 14.025 (10) Å	µ = 9.52 mm^−^^1^
*c* = 7.531 (7) Å	*T* = 291 (2) K
β = 103.212 (2)º	Block, yellow
*V* = 1694 (2) Å^3^	0.10 × 0.10 × 0.10 mm
*Z* = 8	

### Data collection

**Table:**

Bruker SMART CCD area-detector diffractometer	3328 independent reflections
Radiation source: fine-focus sealed tube	1670 reflections with *I* > 2σ(*I*)
Monochromator: graphite	*R*_int_ = 0.109
*T* = 291(2) K	θ_max_ = 26.0º
φ and ω scans	θ_min_ = 1.9º
Absorption correction: multi-scan(SADABS; Bruker, 2000)	*h* = −20→15
*T*_min_ = 0.450, *T*_max_ = 0.450	*k* = −17→16
8777 measured reflections	*l* = −7→9

### Refinement

**Table:**

Refinement on *F*^2^	Hydrogen site location: inferred from neighbouring sites
Least-squares matrix: full	H-atom parameters constrained
*R*[*F*^2^ > 2σ(*F*^2^)] = 0.045	*w* = 1/[σ^2^(*F*_o_^2^) + (0.0451*P*)^2^] where *P* = (*F*_o_^2^ + 2*F*_c_^2^)/3
*wR*(*F*^2^) = 0.106	(Δ/σ)_max_ < 0.001
*S* = 0.79	Δρ_max_ = 0.67 e Å^−^^3^
3328 reflections	Δρ_min_ = −0.55 e Å^−^^3^
202 parameters	Extinction correction: SHELXTL (Sheldrick, 2008), Fc^*^=kFc[1+0.001xFc^2^λ^3^/sin(2θ)]^-1/4^
Primary atom site location: structure-invariant direct methods	Extinction coefficient: 0.0018 (3)
Secondary atom site location: difference Fourier map	

### Special details

**Table:**

Experimental. The structure was solved by direct methods (Bruker, 2000) and successive difference Fourier syntheses.
Geometry. All e.s.d.'s (except the e.s.d. in the dihedral angle between two l.s. planes) are estimated using the full covariance matrix. The cell e.s.d.'s are taken into account individually in the estimation of e.s.d.'s in distances, angles and torsion angles; correlations between e.s.d.'s in cell parameters are only used when they are defined by crystal symmetry. An approximate (isotropic) treatment of cell e.s.d.'s is used for estimating e.s.d.'s involving l.s. planes.
Refinement. Refinement of *F*^2^ against ALL reflections. The weighted *R*-factor *wR* and goodness of fit *S* are based on *F*^2^, conventional *R*-factors *R* are based on *F*, with *F* set to zero for negative *F*^2^. The threshold expression of *F*^2^ > σ(*F*^2^) is used only for calculating *R*-factors(gt) *etc*. and is not relevant to the choice of reflections for refinement. *R*-factors based on *F*^2^ are statistically about twice as large as those based on *F*, and *R*- factors based on ALL data will be even larger.

### Fractional atomic coordinates and isotropic or equivalent isotropic displacement parameters (Å^2^)

**Table:**

	*x*	*y*	*z*	*U*_iso_*/*U*_eq_	
Br1	0.33358 (5)	0.40384 (5)	0.34454 (11)	0.0666 (3)	
Br2	0.32451 (4)	−0.00188 (5)	0.34528 (10)	0.0653 (3)	
Br3	0.10268 (5)	0.96790 (5)	0.39100 (13)	0.0750 (3)	
Br4	−0.12580 (4)	0.67358 (6)	0.46512 (11)	0.0727 (3)	
C1	0.5095 (4)	0.1950 (4)	0.3046 (8)	0.0424 (15)	
C2	0.4701 (4)	0.2838 (4)	0.3143 (8)	0.0455 (16)	
C3	0.3876 (4)	0.2848 (5)	0.3376 (8)	0.0490 (17)	
C4	0.3467 (4)	0.2004 (5)	0.3511 (9)	0.0539 (18)	
H4	0.2925	0.2016	0.3681	0.065*	
C5	0.3858 (4)	0.1130 (5)	0.3396 (8)	0.0504 (17)	
C6	0.4670 (4)	0.1100 (5)	0.3151 (8)	0.0488 (17)	
H6	0.4927	0.0518	0.3059	0.059*	
C7	0.5945 (4)	0.1906 (5)	0.2718 (9)	0.0574 (19)	
H7	0.6177	0.1306	0.2649	0.069*	
C8	0.1215 (4)	0.6721 (5)	0.4036 (9)	0.0494 (17)	
C9	0.1402 (4)	0.7692 (5)	0.3912 (8)	0.0456 (16)	
C10	0.0787 (4)	0.8359 (4)	0.4063 (8)	0.0477 (17)	
C11	0.0001 (4)	0.8089 (5)	0.4295 (9)	0.0542 (18)	
H11	−0.0393	0.8548	0.4395	0.065*	
C12	−0.0188 (4)	0.7110 (5)	0.4376 (9)	0.0510 (18)	
C13	0.0415 (4)	0.6440 (5)	0.4260 (8)	0.0518 (17)	
H13	0.0294	0.5795	0.4329	0.062*	
C14	0.1838 (5)	0.5982 (5)	0.3899 (10)	0.066 (2)	
H14	0.1689	0.5349	0.4011	0.079*	
O1	0.6364 (3)	0.2604 (3)	0.2531 (7)	0.0702 (15)	
O2	0.5114 (3)	0.3678 (3)	0.3080 (7)	0.0655 (13)	
H2	0.5587	0.3568	0.2960	0.098*	
O3	0.2528 (3)	0.6135 (3)	0.3652 (8)	0.0762 (15)	
O4	0.2139 (2)	0.8015 (3)	0.3645 (7)	0.0563 (12)	
H4A	0.2422	0.7563	0.3452	0.084*	

### Atomic displacement parameters (Å^2^)

**Table:**

	*U*^11^	*U*^22^	*U*^33^	*U*^12^	*U*^13^	*U*^23^
Br1	0.0687 (5)	0.0551 (5)	0.0819 (6)	0.0197 (4)	0.0292 (4)	0.0055 (4)
Br2	0.0571 (5)	0.0538 (5)	0.0879 (6)	−0.0152 (4)	0.0226 (4)	−0.0017 (4)
Br3	0.0666 (5)	0.0371 (5)	0.1317 (8)	0.0011 (4)	0.0441 (5)	−0.0014 (5)
Br4	0.0484 (5)	0.0764 (6)	0.0966 (6)	−0.0142 (4)	0.0233 (4)	0.0000 (5)
C1	0.041 (4)	0.039 (4)	0.048 (4)	−0.005 (3)	0.012 (3)	0.000 (3)
C2	0.054 (4)	0.034 (4)	0.049 (4)	−0.005 (3)	0.012 (3)	0.004 (3)
C3	0.048 (4)	0.051 (5)	0.047 (4)	0.010 (3)	0.010 (3)	−0.001 (4)
C4	0.043 (4)	0.060 (5)	0.060 (4)	−0.003 (4)	0.016 (3)	−0.004 (4)
C5	0.050 (4)	0.049 (5)	0.054 (4)	−0.007 (3)	0.015 (3)	−0.008 (4)
C6	0.040 (4)	0.046 (4)	0.060 (5)	−0.003 (3)	0.011 (3)	0.001 (4)
C7	0.050 (4)	0.046 (5)	0.078 (5)	0.008 (3)	0.018 (4)	0.006 (4)
C8	0.053 (4)	0.041 (4)	0.055 (4)	−0.004 (3)	0.014 (3)	0.003 (3)
C9	0.043 (4)	0.046 (4)	0.049 (4)	0.001 (3)	0.011 (3)	−0.001 (3)
C10	0.049 (4)	0.041 (4)	0.055 (4)	−0.002 (3)	0.015 (3)	−0.001 (3)
C11	0.049 (4)	0.056 (5)	0.059 (4)	0.002 (4)	0.016 (3)	−0.005 (4)
C12	0.041 (4)	0.063 (5)	0.052 (4)	0.001 (3)	0.018 (3)	0.007 (4)
C13	0.053 (4)	0.042 (4)	0.062 (5)	−0.010 (3)	0.017 (3)	0.005 (4)
C14	0.077 (6)	0.035 (4)	0.091 (6)	0.013 (4)	0.028 (5)	0.011 (4)
O1	0.049 (3)	0.052 (3)	0.116 (4)	−0.002 (3)	0.032 (3)	0.010 (3)
O2	0.057 (3)	0.045 (3)	0.098 (4)	0.001 (2)	0.026 (3)	0.004 (3)
O3	0.059 (3)	0.049 (3)	0.130 (5)	0.013 (3)	0.043 (3)	0.013 (3)
O4	0.037 (3)	0.041 (3)	0.097 (4)	0.001 (2)	0.029 (2)	−0.002 (3)

### Geometric parameters (Å, °)

**Table:**

Br1—C3	1.898 (6)	C7—H7	0.9300
Br2—C5	1.906 (6)	C8—C9	1.404 (9)
Br3—C10	1.902 (6)	C8—C13	1.424 (8)
Br4—C12	1.895 (6)	C8—C14	1.479 (9)
C1—C6	1.394 (8)	C9—O4	1.355 (7)
C1—C2	1.413 (8)	C9—C10	1.402 (8)
C1—C7	1.477 (8)	C10—C11	1.398 (8)
C2—O2	1.368 (7)	C11—C12	1.412 (9)
C2—C3	1.410 (8)	C11—H11	0.9300
C3—C4	1.377 (8)	C12—C13	1.384 (8)
C4—C5	1.397 (8)	C13—H13	0.9300
C4—H4	0.9300	C14—O3	1.213 (8)
C5—C6	1.393 (8)	C14—H14	0.9300
C6—H6	0.9300	O2—H2	0.8200
C7—O1	1.225 (7)	O4—H4A	0.8200
			
C6—C1—C2	120.5 (6)	C9—C8—C14	120.7 (6)
C6—C1—C7	118.7 (6)	C13—C8—C14	119.4 (6)
C2—C1—C7	120.7 (6)	O4—C9—C10	118.6 (6)
O2—C2—C3	119.8 (6)	O4—C9—C8	123.5 (6)
O2—C2—C1	121.3 (6)	C10—C9—C8	118.0 (6)
C3—C2—C1	118.9 (6)	C11—C10—C9	122.4 (6)
C4—C3—C2	120.2 (6)	C11—C10—Br3	118.9 (5)
C4—C3—Br1	120.9 (5)	C9—C10—Br3	118.7 (5)
C2—C3—Br1	118.9 (5)	C10—C11—C12	119.3 (6)
C3—C4—C5	120.6 (6)	C10—C11—H11	120.4
C3—C4—H4	119.7	C12—C11—H11	120.4
C5—C4—H4	119.7	C13—C12—C11	119.3 (6)
C6—C5—C4	120.3 (6)	C13—C12—Br4	121.2 (5)
C6—C5—Br2	120.5 (5)	C11—C12—Br4	119.6 (5)
C4—C5—Br2	119.1 (5)	C12—C13—C8	121.1 (6)
C5—C6—C1	119.5 (6)	C12—C13—H13	119.4
C5—C6—H6	120.2	C8—C13—H13	119.4
C1—C6—H6	120.2	O3—C14—C8	125.1 (7)
O1—C7—C1	124.5 (6)	O3—C14—H14	117.4
O1—C7—H7	117.7	C8—C14—H14	117.4
C1—C7—H7	117.7	C2—O2—H2	109.5
C9—C8—C13	119.9 (6)	C9—O4—H4A	109.5

### Hydrogen-bond geometry (Å, °)

**Table:**

*D*—H···*A*	*D*—H	H···*A*	*D*···*A*	*D*—H···*A*
O2—H2···O1	0.82	1.94	2.660 (6)	146
O4—H4A···O3	0.82	2.01	2.713 (6)	143
O4—H4A···O1^i^	0.82	2.29	2.863 (6)	128
C7—H7···O3^ii^	0.93	2.55	3.122 (8)	120

Symmetry codes: (i) −*x*+1, *y*+1/2, −*z*+1/2; (ii) −*x*+1, *y*−1/2, −*z*+1/2.
